# Cleft@18–23 study research clinics: a protocol for a multicentre observational study across UK cleft centres to understand variation in outcomes at the end of routine cleft care

**DOI:** 10.1136/bmjopen-2025-106527

**Published:** 2025-11-04

**Authors:** Abhaya Vadlamudi, Stephanie van Eeden, Jenna Spry, Kerry Humphries, Amy Roosbey, Edward Baxter, Sam Leary, Yvonne Wren

**Affiliations:** 1 University of Bristol, Bristol, UK; 2 Newcastle University, Newcastle upon Tyne, UK; 3 University of Exeter, Exeter, UK

**Keywords:** cleft lip, cleft palate, patient reported outcome measures, treatment outcome, clinical protocols, patient satisfaction

## Abstract

**Abstract:**

**Introduction:**

Cleft lip and/or palate (CL/P) is a lifelong condition affecting one in 700 births. In the UK, individuals born with CL/P follow a care pathway at specialist regional cleft centres, which includes input from a range of professionals including surgeons, speech and language therapists, cleft specialist nurses, orthodontists, dentists and clinical psychologists. The cleft centres provide care from diagnosis to early adulthood. Individuals born with CL/P are typically discharged from routine care at their cleft centre between the ages of 15 and 25 years.

Outcome measures of cleft care are currently gathered at different timepoints across the treatment pathway nationally and include outcomes for speech, growth, dental health and psychosocial well-being. However, there is no consistent reporting of outcomes for young adults when they complete routine care, meaning we do not know whether variation in outcomes exists and what this might look like.

This research programme will investigate whether outcomes vary based on factors such as geographical location, biological sex, socioeconomic status or ethnicity. By understanding how outcomes might vary, and the scale and type of variation, we plan to work with young adults born with CL/P and specialist clinicians to develop ways to ensure that everyone born with CL/P in the UK receives the optimum care to meet their needs.

**Methods and analysis:**

Cleft@18–23 is an observational study of young adults born with CL/P. Recruitment is planned across all regions of the UK, beginning in April 2025 with research clinics scheduled to run between June 2025 and May 2027. The recruitment target is 640 participants born with CL/P. Participants with all cleft diagnoses, including those with additional syndromic diagnoses, will be eligible for recruitment. We will recruit participants from all ethnic and socioeconomic backgrounds. Data collection will include self-report participant questionnaires, speech samples, a hearing screen, two-dimensional and three-dimensional medical photographs, an intraoral scan and a dental assessment. A range of descriptive and inferential statistical analyses will explore variation in outcomes across different groups.

**Ethics and dissemination:**

The Cleft@18–23 study obtained ethical approval from the South West-Frenchay Research Ethics Committee on 26 November 2024 (REC reference: 24/SW/0128). Informed consent will be required for participation. Findings from the Cleft@18–23 study will be disseminated through peer-reviewed publications, conference presentations, newsletters, the study website (https://www.bristol.ac.uk/cleft18-23) and social media.

**Trial registration number:**

ISRCTN34027276.

**STRENGTHS AND LIMITATIONS OF THIS STUDY**

- First known dataset charting multidisciplinary outcomes at end of routine care after centralisation of cleft care in the UK.
- Nationwide study involving people born with all cleft subtypes including additional diagnoses.
- Strong collaboration with all key stakeholders including young adults with lived experience of cleft, clinicians from all professions and the charity Cleft Lip and Palate Association.
- Potential selection bias through difficulty reaching some underserved communities and only considers people who have had all cleft care in the UK.
- Ability to attend long appointments for young working adults may limit participation.

## Introduction

 Cleft lip and/or palate (CL/P) is one of the most common congenital anomalies, affecting over 1000 babies in the UK each year.^1^ People born with CL/P can experience difficulties with appearance, speech, language, hearing, dental health, facial growth and psychosocial well-being. Treatment for this population needs to be timely and accessible. Despite centralisation of cleft care in the UK over two decades ago, variation in accessing this care is reported, leading to health inequalities across different regions and socioeconomic and ethnic groups.^2 3^

Primary surgery takes place in the first year of life, with repair of a cleft lip and hard palate between 3 and 6 months of age and a repair of the soft palate between 9 and 12 months of age.^4^ On average, children born with CL/P have 3.2 admissions and spend 13.2 days in hospital in the first 2 years of life. This can range from 2.6 admissions and 9.2 days for children born with cleft lip only to 4.7 admissions and 19.7 days in children born with a bilateral cleft lip and palate.^5^ Thereafter, these children attend multiple clinical appointments with a multidisciplinary team comprising speech and language therapists, audiologists, paediatric dentists, specialist orthodontists and restorative dentists, clinical psychologists, cleft nurse specialists and surgeons for approximately 20 years. Once a young adult with CL/P has completed scheduled interventions (typically between the ages of 15 and 25 years), they transition from a care pathway facilitated via parents, structured around routine appointments and regular follow-up to a system of on-request care driven by the young person themself.

In two focus groups, 10 young adults born with CL/P aged 16–20 years reported that they found this transition in responsibility for, and ownership of care, difficult to navigate. The focus groups revealed that the transition coincides with other challenges to mental health and self-confidence as individuals start work or move away from home.^6^ The young adults added that concerns about appearance can increase in young adulthood together with worries about speech and hearing, sometimes leading to feelings of isolation.

UK cleft teams have acknowledged that variation exists in services and have suggested that outcomes for young adults born with CL/P at transition may differ between subpopulations, including those identified by equality, diversity and inclusion characteristics. Specifically, concerns about variation due to geography, ethnicity and socioeconomic status were raised. The focus group with young adults born with CL/P identified concerns that young men were less likely to access support than young women.

The Cleft@18–23 study therefore aims to be the first to investigate outcomes for young people born with CL/P after completion of routine care and explore and explain any observed variation. It will also seek to develop a tool to help all young people navigate the transition of care and access ongoing treatment and support as adults where necessary. The study programme will achieve this through four work packages. The first of these work packages (WP1) is the Cleft@18–23 research clinics. The focus of this work package is to collect data on outcomes at the end of routine care and to explore variations across regions, socioeconomic status, ethnicity, gender and biological sex. The protocol for WP1 is presented in this paper.

The WP1 protocol has been developed with input from a series of focus groups with each of the core disciplines involved in cleft care (speech and language therapists, audiologists, dentists including specialist orthodontists and restorative dentists, clinical psychologists, cleft nurse specialists and surgeons) and two focus groups with young people aged 16–20 years born with a CL/P. The protocol was further refined with input from all 16 regional centres following visits to each centre by the Chief Investigator, and by patient and public involvement (PPI) partners in this programme of work.

This study aims to answer the following primary research question: what are the levels of emotional well-being in young adults aged 18–23 years born with a CL/P and do these vary across ethnicity, biological sex, socioeconomic status and geographic location?

## Methods and analysis

### Study design and setting

**Figure 1:**
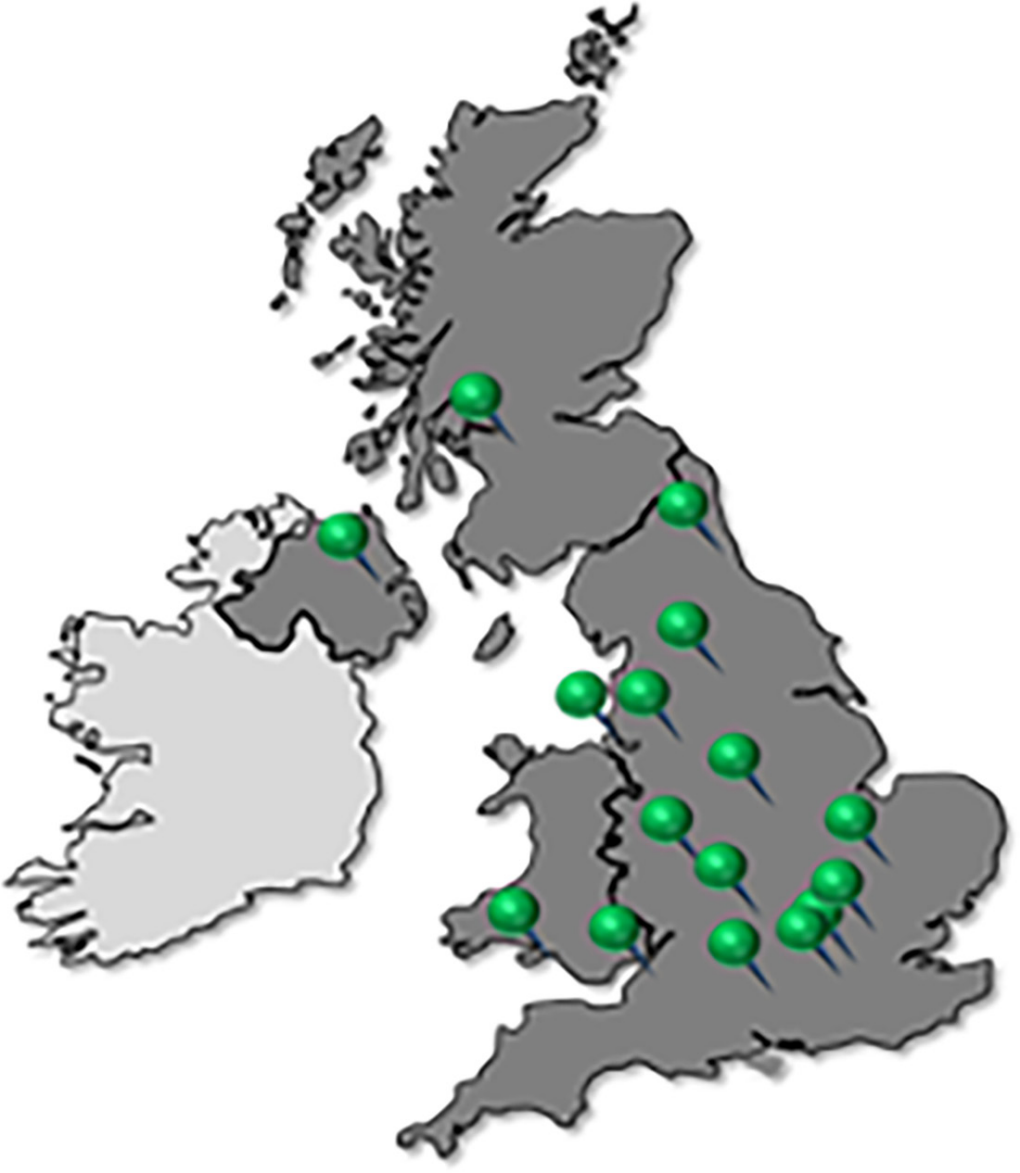
Map showing locations of all UK regional cleft centres.

The Cleft@18–23 research clinic work package is a multicentre observational study. Research clinics will take place across sites covering the 16 regional cleft units in the UK (figure 1).

### Study population

Participants will be aged 18–23 years and will have been discharged from the full cleft multidisciplinary service at least 6 months prior to taking part in this study. They will no longer be receiving any active treatment that impacts outcomes such as appearance, speech and well-being but may be under review (eg, for refitting of retainers or for advice from genetic counselling). To be eligible for the study, participants must have had their primary cleft surgery in the UK before the age of 2 years and have continued their care entirely within the UK. Participants born with all cleft subtypes, including those with and without additional syndromic diagnoses, will be eligible to take part. The age group 18–23 years was chosen to ensure a sample population reflecting postcentralisation of cleft care in the UK is achieved.^7^

### Power calculations

A sample of 600 would allow a mean score of 86.4 to be estimated with a 95% CI of 84.2 to 88.6 (mean and SD based on Foo *et al*^8^) for the primary outcome measure (the emotional role subscale of Short Form 36 (SF-36)). With just over 1000 babies born each year with CL/P, the total population available to recruit from over a 2-year period is approximately 7000 (1000 individuals born with cleft each year for ages 18–23—totalling 6000—plus an additional year of recruitment to include young adults who turn 18 during the first year of recruitment). The recruitment rate for a comparable study run by the same team and involving younger children was 75%^9^; therefore, recruiting 600 is achievable. Nevertheless, we propose to over-recruit by 40 (total sample size 640) as contingency for missing data.

To achieve a sample size of 640, we aim to recruit from across the UK’s cleft teams. Some serve a larger population than others, so it is likely that we may recruit more from some sites than others. Our work with young people to date suggests that they would be willing to attend this research clinic. We aim to recruit participants across all groups to ensure that the study sample reflects the general population in terms of ethnicity, socioeconomic status, gender and cleft subtypes. Recruitment will be facilitated through targeted dissemination of study information via general medical practices, dental practices and community organisations serving diverse ethnic groups. Regular evaluation of those recruited will ensure the sample is representative of target population in ethnicity, biological sex, socioeconomic status and geographic location to minimise the risk of selection bias.

### Recruitment and consent

**Figure 2:**
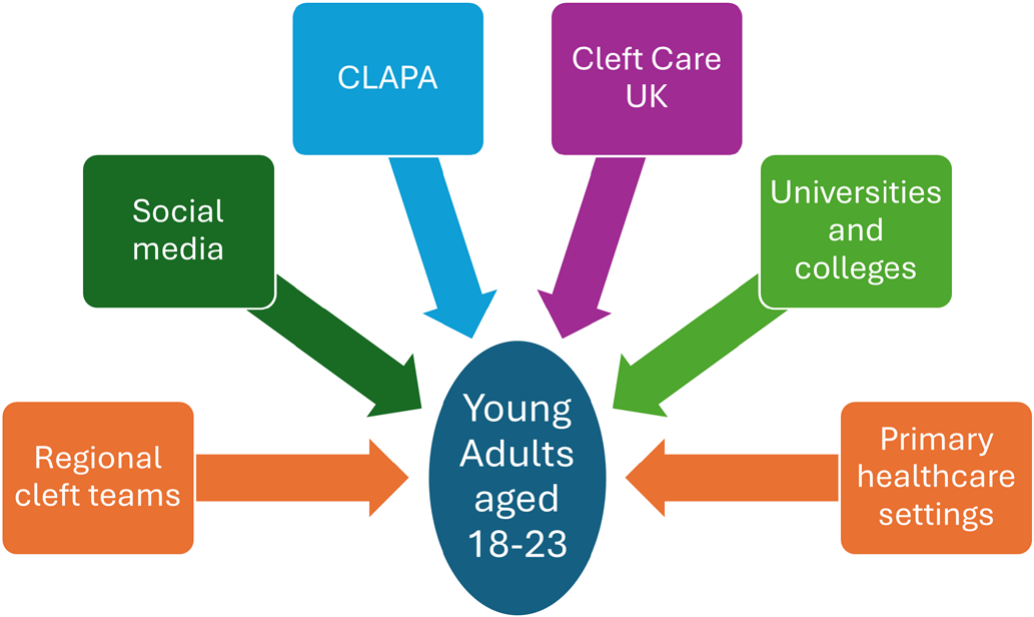
The six routes to recruitment for the Cleft@18–23 study. CLAPA, Cleft Lip and Palate Association.

Recruitment began in April 2025, with data collection planned to end in May 2027. Potential participants will be made aware of the study through one of six routes (figure 2). These include contact by their local cleft teams or the Cleft Lip and Palate Association (CLAPA) (https://www.clapa.com), contacting families who participated in the Cleft Care UK research project and indicated an interest in further research,^9^ and promoting the study through universities, colleges and primary healthcare providers such as dental practices and general practitioner surgeries. The study will also be promoted via social media with the help of the PPI group (see figure 2 Figure 2).

Each of these routes will provide a link to the study website where more information is available and where there is an option to complete an initial ‘Expression of Interest’ contact form, which will automatically be sent to the study team. The form will help the study team determine eligibility. If a potential participant is eligible, they will be contacted by a member of the study team who has completed Good Clinical Practice training. They will explain the research clinic and go through the consent form. A link to an electronic consent form will be sent to the participant and this will be checked at the research clinic by a member of the study team. Paper consent forms will be available on request. Those who register their interest and are not eligible to take part will be directed to a form to provide their contact details if they are interested in finding out about any future opportunities.

### Procedures

**Figure 3:**
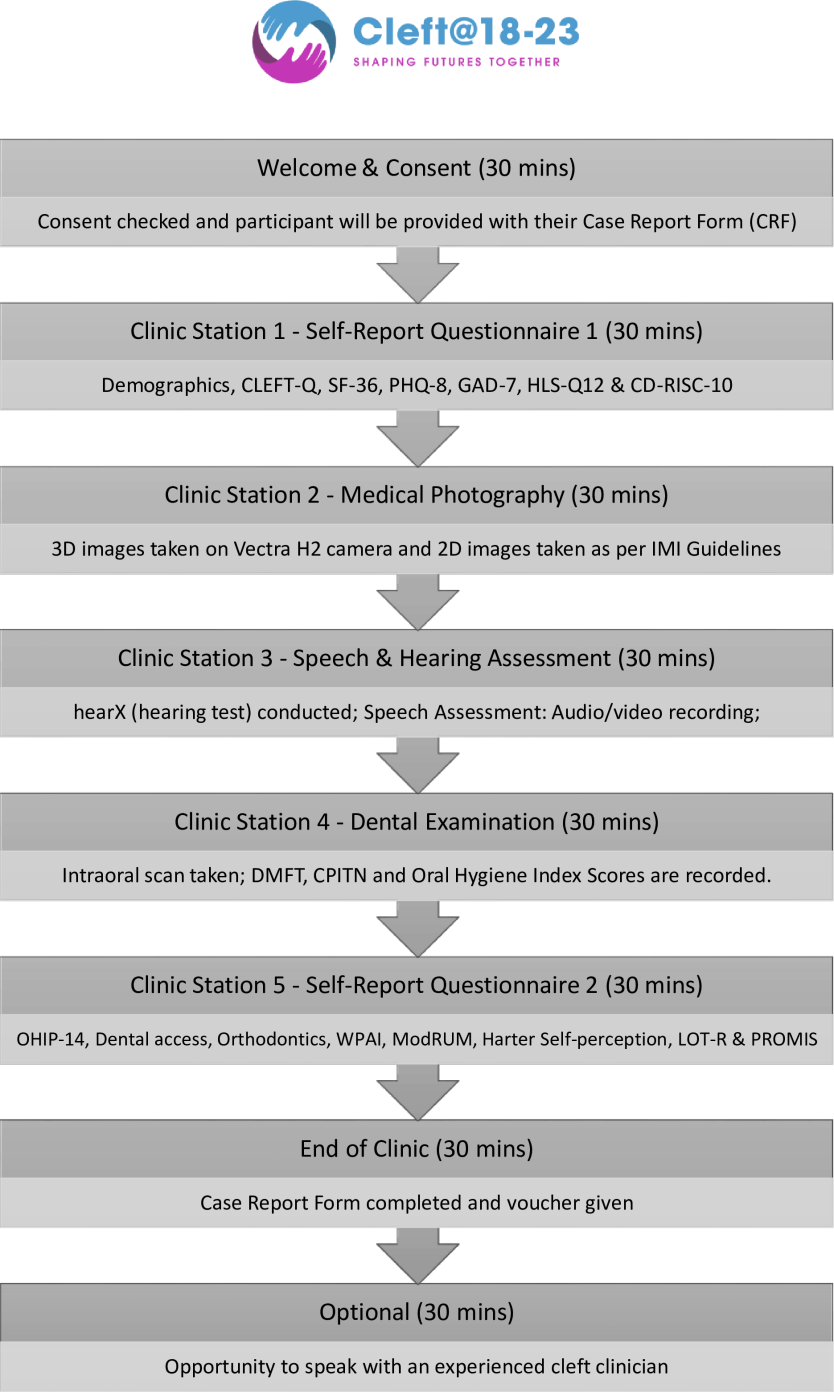
Flow chart of research clinic activity. 2D, two-dimensional; 3D, three-dimensional; CD-RISC-10, Connor-Davidson Resilience Scale-10; CLEFT-Q, Cleft Questionnaire; CPITN, Community Periodontal Index for Treatment Needs; DMFT, Decayed, Missing, Filled Teeth Index; GAD-7, Generalised Anxiety Disorder-7; HLS-Q12, Health Literacy Survey-Q12; IMI, Institute of Medical Illustrators; LOT-R, Revised Life Orientation Test; ModRUM, MODular Resource-Use Measure; OHIP-14, Oral Health Impact Profile-14; PHQ-8, Patient Health Questionnaire-8; PROMIS, Patient-Reported Outcome Measurement Information System; SF-36, Short Form 36; WPAI, Work Productivity and Activity Impairment questionnaire.

Participants will attend a research clinic as close to home as possible. The clinics will last approximately 3 hours. During the research clinics, participants will be guided through five stations, which are outlined in figure 3.

### Data collection methods

Data will be collected through self-report questionnaires, photographic images, hearing and speech assessments and a dental examination (including an intraoral scan). The research clinic will be facilitated by a member of the Cleft@18–23 team with support from an administrative personnel member from the local site team. All data collected from the research clinics are summarised in table 1.

**Table 1:** Summary of research clinic stations and data collection

Research clinic station	Measures	Onsite activity for data collection
1. Self-report questionnaire—part 1	Demographics including EDI characteristics	Self-report
	Satisfaction with appearance and function	CLEFT-Q
	Health-related quality of life (emotional role subscale) (*PRIMARY OUTCOME MEASURE*)	SF-36
	Depression	PHQ-8
	Anxiety	GAD-7
	Health literacy	HLS-Q12
	Resilience	CD-RISC-10
2. Medical photography	Nasolabial appearance/teeth appearance	2D photographs
	Facial shape and symmetry	3D images
3. Hearing and speech assessment	Hearing	Pure tone audiometry
	Speech	Audio-visual recording of a speech sample
4. Dental examination	Dental arch alignment	Intraoral scans
	Oral health—dental	DMFT
	Oral health—periodontal	CPITN and OHI-S
5. Self-report questionnaire—part 2	Satisfaction with oral health	OHIP-14
	Access to treatment	Dental access self-report
	Orthodontic treatment	Orthodontic treatment self-report
	Productivity loss	WPAI
	Resource use	ModRUM
	Use of private healthcare	Private healthcare use self-report
	Out-of-pocket expenses	Out-of-pocket expenses self-report
	Social functioning	Harter self-perception profile
	World view	LOT-R
	Global health-related quality of life	PROMIS

CD-RISC-10, Connor-Davidson Resilience Scale-10; CLEFT-Q, Cleft Questionnaire; CPITN, Community Periodontal Index for Treatment Needs; 2D, two-dimensional; 3D, three-dimensional; DMFT, Decayed, Missing, Filled Teeth Index; EDI, Equality, Diversity and Inclusion; GAD-7, Generalised Anxiety Disorder-7; HLS-Q12, Health Literacy Survey-Q12; LOT-R, Revised Life Orientation Test; ModRUM, MODular Resource-Use Measure; OHIP-14, Oral Health Impact Profile-14; OHI-S, Oral Hygiene Index-Simplified; PHQ-8, Patient Health Questionnaire-8; PROMIS, Patient Reported Outcome Measurement Information System; SF-36, Short Form 36; WPAI, Work Productivity and Activity Impairment questionnaire.

#### Self-report questionnaires

The self-report questionnaires omprise questions relating to demographics and experience along with measures from validated patient questionnaires, which are discussed further under the ‘Derived variables’ section. The self-report questionnaire will be presented electronically through Research Electronic Data Capture (a secure web application for building and managing online surveys and database: https://project-redcap.org) and Concerto (an adaptive testing platform developed and maintained by the University of Cambridge Psychometrics Centre: https://concertoplatform.com). The self-report questionnaire will be broken down into two parts: the first part of the questionnaire to be completed at the beginning of the research clinic and the second part towards the end of the research clinic. This is based on input from PPI to split the questionnaire into two parts for participants’ ease. If a participant has learning difficulties, a friend, carer, parent or member of the study team will be able to help read the questions and record their responses. A summary of the measures used in each section of the self-report questionnaires is provided in table 1. The first part of the questionnaire will also collect data on general demographics including age (in years), sex at birth, gender, ethnicity, parental employment (as an indicator of socioeconomic status), cleft subtype, syndromic status and cleft surgical history (number and type of operations), which will be used as the exposures/confounders/effect modifiers in statistical analysis. In the second part of the questionnaire, further questions will collect data on access to dental care and experience of orthodontic treatment; these have been developed by the cleft specialist dentists and orthodontists in the UK. Following recommendations from our co-applicants and PPI group, the end of each part of the self-report questionnaire concludes with optional questions, which focus on how having a cleft may have had a positive impact on participants. This section will comprise a series of statements that the participant can opt to complete:

- Advice I would give my younger self…
- I’m proud of myself because…
- Something I want people to know about me is…

#### Medical photography

A medical photographer will take 2D and 3D images, following the Institute of Medical Illustrators guidelines.^10^ Both core and optional 2D views will be captured. These include extraoral (lateral, anterior-posterior views of the face and the lip and nose areas) and intraoral views (buccal teeth, palate, occlusal mirror, teeth mirror, teeth overjet and alveolus). The Vectra H2 3D camera will capture 3D views from three angles: 45° left, front and 45° right of the face.

#### Hearing

Hearing levels will be screened by the speech and language therapist facilitating the research clinic using the hearScreen from hearX.^11^ This provides a reading for each ear across a range of frequencies using calibrated over-ear headphones and secure software via a Samsung Galaxy smartphone. It takes 5–10 min and has been validated for use outside of audio booths.^12^ Participants will be instructed by the speech and language therapist to place the headphones over their ears. The speech and language therapist will play tones via the smartphone at a 35 dB level. The participant will indicate when they have heard a tone by raising their hand and the speech and language therapist will record this via the smartphone.

#### Speech

A standardised speech sample will be collected using high-quality audio and video recordings in a quiet room with the subject facing natural light if possible. Each participant will sit in front of a pale neutral background. The face and upper neck will be framed in the picture. The sample will follow the protocol used in the Great Ormond Street Speech Assessment (GOS.SP.ASS)^13^ and includes the following:

- Conversational speech.
- Rote speech including counting from 1 to 20 and 60 to 70.
- Reciting the days of the week.
- Repetition of the 16 GOS.SP.ASS sentences.
- A picture description task for connected speech and language sample.

#### Dental examination

The study dental therapist/hygienist will carry out the dental examination. They will check for decayed, missing and filled teeth and carry out a periodontal examination. A digital impression for dental arch alignment will be made using the Dentsply Sirona Primescan two digital intraoral scanner, which will be used to take occlusal, buccal, lingual, approximal and full jaw scans. Bite is also captured. The scanner will be used, cleaned and disinfected, in accordance with the guidance provided by the manufacturer (Dentsply Primescan Connect Operating Instructions, available from https://www.dentsplysirona.com/en-gb/discover/discover-by-brand/primescan.html). The digital scans will be captured on a study laptop used specifically for the collection of intraoral scans.

### Derived variables

#### Primary outcome measure

The variable used as the primary outcome measure for this study will be derived from the SF-36.^14^ We will use the five questions of the emotional role subscale of SF-36, also known as Mental Health Inventory. SF-36 was chosen to derive the primary outcome measure following the focus groups held with young adults born with CL/P,^6^ during which it was established that emotional well-being was the primary concern while transitioning into adult care.

The Patient-Reported Outcome Measurement Information System (PROMIS)^15^ Global Health Scale will also be included. This is a standardised psychometric instrument containing 10 questions pertaining to health-related quality of life. The SF-36 emotional role subscale will remain as the primary outcome measure due to its use in calculating the power of the study and for comparison with older literature.

The decision to retain both the SF-36 and the PROMIS, which has been designed to supersede the SF-36, was driven by the desire to compare the results from this study with other studies, which were carried out prior to the development of the PROMIS and which therefore used the SF-36. However, we also want to enable future researchers to compare their results using the PROMIS with our data and using only the SF-36 would limit that ability. In order to reduce repetition within this part of the questionnaire, we limited the questions from the SF-36 to just those five which comprise the emotional role subscale, as the study was designed with these forming the primary outcome measure and the means for calculating the sample size needed to ensure a robustly powered study.

#### Secondary outcome measures

There are multiple secondary outcome measures for this study covering the domains of patient satisfaction, aspects of psychosocial well-being, facial appearance, hearing and speech, dental outcomes and oral health, growth, access to care and the economic impact of a cleft diagnosis for patients. The derived variables for these are outlined below.

##### Satisfaction with appearance and function

The Cleft Questionnaire^16^ is a standardised patient-reported outcome measure specific to the CL/P population suitable for use with people aged 8–29 years. Variables reflecting patient satisfaction with the following will be derived:

- Appearance of the face
- Appearance of the lips
- Appearance of the nose
- Appearance of the nostrils
- Appearance of the jaws
- Appearance of the teeth
- Appearance of the cleft lip scar
- Psychological function
- Social function
- Speech distress
- Speech function
- Eating and drinking

Additional variables regarding patient satisfaction with oral health will be collected using the Oral Health Impact Profile-14. This is a 14-item questionnaire used to measure participants’ satisfaction with their teeth, mouth or dentures.^17^ This will allow comparisons with normative data from the general population.

##### Psychosocial well-being

Variables pertaining to the presence and severity of depression and anxiety will be derived from the Patient Health Questionnaire-8^18^ and the Generalised Anxiety Disorder-7.^19^ A variable measuring participants’ sociability and relationships will be derived from the Harter Self-Perception profile.^20^ A variable measuring resilience in stressful situations will be derived from the Connor-Davidson Resilience Scale-10 (CD-RISC-10), a 10-item scale derived from the 25-item CD-RISC-25, will be used to measure ability to cope with stressful life situations.^21^

##### Facial appearance

Variables measuring nasolabial appearance will be derived from the Asher McDade Index^22^ and variables measuring facial shape and symmetry from data generated from the software from the Vectra H2 3D camera.^23^

##### Hearing and speech

Data from a hearing screen will indicate hearing acuity for each ear at the frequencies 500 Hz, 1000 Hz, 2000 Hz, 4000 Hz, 8000 Hz. A speech and language sample will provide data on ongoing velopharyngeal function and speech sound errors. After the research clinics, two specialist cleft speech and language therapists will carry out consensus listening of the speech samples and rate them following the Cleft Audit Protocol for Speech-Augmented (CAPS-A).^24 25^ The CAPS-A analysis will generate data from which we will create the following variables for statistical analysis:

- Intelligibility on a 5-point scale (normal, different from other people’s speech but not enough to cause comment, different enough to provoke comment, but possible to understand most speech, only just intelligible to strangers, impossible to understand).
- Velopharyngeal composite score on a 3-point scale (competent, marginally incompetent, incompetent).^26 27^
- Absence/Presence of active cleft speech characteristics in three categories (anterior, posterior, non-oral).
- Per cent Consonants Correct measure (continuous variable).

##### Dental outcomes and oral health

Dental health will be measured using the sum of the number of Decayed, Missing and Filled Teeth^28^ in the permanent teeth as the standard protocol for measurement of dental caries. A further calculation of decayed and filled teeth divided by the total number of teeth) will be calculated to derive a score, which will assess dental health based on decay, given that many teeth are missing for reasons other than decay in individuals born with CL/P. A variable regarding periodontal health will be obtained from the Community Periodontal Index for Treatment Needs^29^ and the simplified Oral Hygiene Index.^30^

##### Growth

The intraoral scanner will provide data on dental arch alignment. Variables will be derived from data generated from analysis of the scans using the Modified Huddart-Bodenham Index.^31^ Peer Assessment Rating scores will also be obtained.^32^

##### The economic impact of a cleft diagnosis for patients

A variable to measure productivity will be derived from the Work Productivity and Activity Impairment questionnaire,^33^ a 6-item questionnaire measuring the effect of participants’ health problems on their ability to work and perform regular activities. Measures of healthcare resource use will be gathered from the MODular Resource-Use Measure.^34^ This is a questionnaire used to measure participants’ use of healthcare resources over the previous 6 months. We will also ask about the use of private healthcare and about any out-of-pocket expenses they may have had related to their cleft healthcare and postsecondary education (if applicable) in the last 6 months.

### Confounders

Health literacy is an important confounder as it will impact participants’ ability to respond to the self-report questionnaires. This will be measured using the Health Literacy Survey-Q12, a 12-item adapted short form questionnaire of the HLS19-Q47 for measuring general health literacy in general adult populations.^35^

The Revised Life Orientation Test will be used to measure the participants’ outlook and world view; this measure of dispositional optimism could influence their satisfaction scores.^36^

### Data analyses

Descriptive statistics will be used to summarise the exposures (ethnicity, sex, gender, socioeconomic status and geographical location) using frequencies and percentages. For the numerical outcomes, central tendency and variation will be summarised by means and SD or medians and IQRs as appropriate. Categorical outcomes will be summarised by frequencies and percentages.

Regression models will be used to assess associations between the exposures and the primary outcome. Secondary analysis will be based on regression models for each of the other outcomes separately. Linear, logistic, ordinal logistic or Poisson regression will be used to model associations for continuous, binary, ordinal or discrete outcomes, respectively; continuous outcomes will be transformed before analysis if model residuals are skewed.

Directed acyclic graphs will be used to conceptualise associations between variables to enable an appropriate set of confounders to be selected for modelling each exposure-outcome association; age and the other exposures will be considered as potential confounders as well as health literacy and dispositional optimism. All models will be fitted before and after adjustment for appropriate confounders. A sensitivity analysis will be undertaken, whereby all unadjusted models will be repeated, restricting to only those with full confounder information available. This will confirm whether any changes after adjustment are due to missing data or the confounding variables themselves.

Potential cleft centre-level variation for each outcome will be identified through estimation of variance partition coefficients from multilevel models with centre treated as a random effect, adjusting for appropriate fixed effects (eg, age and sex). Any outcomes displaying centre-level variation will be modelled using multilevel rather than single-level models.

Potential effect modification through cleft subtype and/or syndromic status will be tested through inclusion of interaction terms in models; if the exposure-outcome association differs according to the categories of cleft subtype/syndromic status, category-specific regression estimates will be presented.

The economic impact of a cleft diagnosis will be collected through the self-report questionnaire and will be analysed to determine variation in resource use within the sample.

### Data deposition and curation

Data are held on a combination of restricted access network folders and the University of Bristol (UoB) servers. All of the above rely on Windows authentication administered by the UoB IT Services and have been approved for storage of this type of data. Data generated by the Cleft@18–23 study will be maintained on secure servers at UoB, which are backed up on a regular basis.

Primary source material (eg, questionnaires, case report forms and consent forms) will be preserved as electronic (scanned) and hard (paper) copies where practicable. Paper copies of consent forms, case report forms and questionnaires will be stored in locked cabinets in restricted access offices until they are digitised. Once digitised, these documents will be securely destroyed in line with institutional policies. Data transfers between systems and collaborators will use encrypted methods (eg, password-protected ZIP files and Advanced Encryption Standard-256 encryption) to minimise the risk of breaches.

At the end of the study, all data will be stored on the UoB Research Data Storage Facility. Where participants have consented, their data will be stored indefinitely and made available to external researchers following the process, which will be detailed in a data access policy. This will include the need for submission of a proposal for use of the data, ethical approval for the analysis planned and approval from the data custodians. We will follow the standards and policies set out by UoB. Further information on data security can be found here: http://www.bristol.ac.uk/infosec/policies/.

### Patient and public involvement

Focus groups with members of the patient community, specifically 10 young adults aged between 16 and 20 years informed the development of the funding application. A PPI group comprising young adults aged 18–30 years born with a CL/P has now been established for the study and this group has informed key elements in the writing of the protocol and development of recruitment resources. We have an ongoing process for recruitment to the PPI group to ensure that we have sufficient PPI representation throughout the study.

PPI has informed:

- the acceptability of the research;
- design of the research.

PPI will have an ongoing role in providing input to:

- recruitment
- management of the research;
- undertaking the research;
- analysis of results;
- dissemination of findings.

### Ethics and dissemination

Ethical approval for Cleft@18–23 was provided by the South West-Frenchay Research Ethics Committee on 26 November 2024 (REC reference: 24/SW/0128). Subsequent Health Research Authority and Health Care Research Wales approval for the study was also given on the same day. Cleft@18–23 is sponsored by University Hospitals Bristol and Weston NHS Foundation Trust. Informed consent is required for participation.

Findings from Cleft@18–23 will be disseminated through peer-reviewed publications and through national and international conference presentations, both oral and poster. To promote open research and support rapid dissemination, the study team and collaborators using the Cleft@18–23 resource for their research may also publish preprints of their papers using preprint servers such as Medrxiv or Biorxiv subject to their target journal guidance. Papers published will be promoted through the Cleft@18–23 website (https://www.bristol.ac.uk/cleft18-23), newsletters and through social media (Bluesky: @cleft1823study.bsky.social; Facebook: cleft1823; Instagram: @cleft1823study; LinkedIn: Cleft1823study).
