# Supplementary material for: Cleft@18–23 study research clinics: a protocol for a multicentre observational study across UK cleft centres to understand variation in outcomes at the end of routine cleft care
Source: BMJ Open. 2025 Nov 4;15(11):e106527. doi: 10.1136/bmjopen-2025-106527 (PMC12587910; doi:10.1136/bmjopen-2025-106527)
Supplement: Supplementary file 1 [file bmjopen-2025-106527.reviewer_comments.pdf]

PEER REVIEW HISTORY

BMJ Open publishes all reviews undertaken for accepted manuscripts. Reviewers are asked to complete a checklist review form and are provided with free text boxes to elaborate on their assessment. These free text comments are reproduced below.

ARTICLE DETAILS

Title (Provisional)

Title: The Cleft@18-23 Study Research Clinics: a protocol for a multicentre observational study across UK cleft centres to understand variation in outcomes at the end of routine cleft care

Authors

Vadlamudi, Abhaya; van Eeden, Stephanie; Spry, Jenna; Humphries, Kerry; Roosbey, Amy; Baxter, Edward; Leary, Sam; Wren, Yvonne

---

VERSION 1 - REVIEW

**Reviewer**	**1**
**Name**	**Ruslin, Muhammad**
**Affiliation**	**Hasanuddin University, Oral and Maxillofacial Surgery**
**Date**	**07-Aug-2025**
**COI**	**None**

Manuscript title: The Cleft@18-23 Study Research Clinics: a protocol for a multicentre cross-sectional cohort study across UK cleft centers.

This article presents a timely and compelling examination of outcome variables in relation to factors such as geographical location, biological sex, socioeconomic status, and ethnicity. I recommend accepting the study with minor revisions.

The following weaknesses in this study and manuscript require further attention:

1. Abstract
   Make sure to explain the study design in the abstract and specify the exact timeframe, including the start and end dates.
2. Introduction
   Could be benefit from a clearer articulation of the primary research question at the end of the introduction
3. Study design and methodology
   - The SPIRIT and STROBE checklists for observational studies are not included but should be provided as supplementary materials as required by BMJ.

- To enhance readability, certain parts of the procedure section could be streamlined or arranged in a bullet or flow format.

4. Outcome measures
   - The rationale for the concurrent use of SF-36 and PROMIS could be articulated more succinctly.
   - A flowchart outlining the study procedures and data points could enhance the manuscript.
5. Sample size and statistical consideration
   The statistical plan for data analysis could benefit from more detail, particularly in terms of addressing missing data, conducting subgroup analyses, and considering potential confounding variables.
6. Ethical and dissemination plan
   Ensure actual name of ethics board and approval number provided before publication
   Include the data availability statement and indicate where data will be stored or shared, per BMJ policy.

VERSION 1 - AUTHOR RESPONSE

	Reviewer comments	Our response
1	**Abstract:** Make sure to explain the study design in the abstract and specify the exact timeframe, including the start and end dates.	This is described in the **Methods and analysis** section of the abstract.
2	**Introduction:** Could be benefit from a clearer articulation of the primary research question at the end of the introduction	The primary research question is now added to the end of the introduction.
3	**Study design and methodology:** The SPIRIT and STROBE checklists for observational studies are not included but should be provided as supplementary materials as required by BMJ.	In progress.
4	**Study design and methodology:** To enhance readability, certain parts of the procedure section could be streamlined or arranged in a bullet or flow format.	We swapped Figure 3 from the previously submitted image to a clinic flowchart which we hope will help clarify this.
5	**Outcome measures:** The rationale for the concurrent use of SF-36 and PROMIS could be articulated more succinctly.	Wording has been added to explain this more clearly.
6	**Outcome measures:** A flowchart outlining the study procedures and	We added clinic flowchart to procedures sections. Data points have been included in Table 1.

	data points could enhance the manuscript.	
7	**Sample size and statistical consideration:** The statistical plan for data analysis could benefit from more detail, particularly in terms of addressing missing data, conducting subgroup analyses, and considering potential confounding variables.	Two sentences explaining how missing data will be managed have now been added to the end of paragraph 3 in the Data analyses.<br><br>There is already a paragraph on potential confounding variables: (*Directed acyclic graphs (DAGs) will be used to conceptualise associations between variables to enable an appropriate set of confounders to be selected for modelling each exposure-outcome association; age and the other exposures will be considered as potential confounders as well as health literacy and dispositional optimism. All models will be fitted before and after adjustment for appropriate confounders)*, as well as a paragraph on subgroup analyses (*Potential effect modification through cleft subtype and/or syndromic status will be tested through inclusion of interaction terms in models; if the exposure-outcome association differs according to the categories of cleft subtype/syndromic status, category-specific regression estimates will be presented.*)
8	**Ethical and dissemination plan:** Ensure actual name of ethics board and approval number provided before publication<br>Include the data availability statement and indicate where data will be stored or shared, per BMJ policy.	We have removed the anonymised sections and added the name of the ethics board and approval number in the revised submission.<br><br>Data availability is described on page 17 under **Data deposition and curation.** We are happy to move this to dissemination if preferred by the reviewer.
